# Verification of P-Glycoprotein Function at the Dermal Barrier in Diffusion Cells and Dynamic “Skin-On-A-Chip” Microfluidic Device

**DOI:** 10.3390/pharmaceutics12090804

**Published:** 2020-08-25

**Authors:** Ágnes Bajza, Dorottya Kocsis, Orsolya Berezvai, András József Laki, Bence Lukács, Tímea Imre, Kristóf Iván, Pál Szabó, Franciska Erdő

**Affiliations:** 1 Faculty of Information Technology and Bionics, Pázmány Péter Catholic University, Práter u. 50a, H-1083 Budapest, Hungary; agnesbajza@hotmail.com (Á.B.); kocsis.dorottya.1@hallgato.ppke.hu (D.K.); kisberezvai@gmail.com (O.B.); laki.andras.jozsef@itk.ppke.hu (A.J.L.); ivan.kristof@itk.ppke.hu (K.I.); 2 Faculty of Chemical Technology and Biotechnology, Budapest University of Technology and Economics, Műegyetem rkp. 3., H-1111 Budapest, Hungary; 3 Department of Biophysics and Radiation Biology, Semmelweis University, Tűzoltó u. 37-47, H-1094 Budapest, Hungary; 4 MedRes Medical Research Engineering Ltd., Albert Flórián út 3/b, H-1097 Budapest, Hungary; lukacs.bence.991@gmail.com or; 5 Research Centre for Natural Sciences, Instrumentation Centre, Magyar tudósok körútja 2, H-1117 Budapest, Hungary; imre.timea@ttk.mta.hu (T.I.); szabo.pal@ttk.mta.hu (P.S.)

**Keywords:** P-glycoprotein, skin-on-a-chip, microfluidic device, Franz diffusion cell, skin permeability, transdermal microdialysis, erythromycin, quinidine, valspodar, dermal barrier

## Abstract

The efficacy of transdermal absorption of drugs and the irritation or corrosion potential of topically applied formulations are important areas of investigation in pharmaceutical, military and cosmetic research. The aim of the present experiments is to test the role of P-glycoprotein in dermal drug delivery in various ex vivo and in vitro platforms, including a novel microchip technology developed by Pázmány Péter Catholic University. A further question is whether the freezing of excised skin and age have any influence on P-glycoprotein-mediated dermal drug absorption. Two P-glycoprotein substrate model drugs (quinidine and erythromycin) were investigated via topical administration in diffusion cells, a skin-on-a-chip device and transdermal microdialysis in rat skin. The transdermal absorption of both model drugs was reduced by P-glycoprotein inhibition, and both aging and freezing increased the permeability of the tissues. Based on our findings, it is concluded that the process of freezing leads to reduced function of efflux transporters, and increases the porosity of skin. P-glycoprotein has an absorptive orientation in the skin, and topical inhibitors can modify its action. The defensive role of the skin seems to be diminished in aged individuals, partly due to reduced thickness of the dermis. The novel microfluidic microchip seems to be an appropriate tool to investigate dermal drug delivery.

## 1. Introduction

Our largest organ, the skin, is a place for the application of cosmetics and also drugs for different therapeutic purposes (both local and systemic). The skin is a defensive organ, protecting against the invasion of foreign substances from the environment to the body and maintaining homeostasis. There are different reasons to monitor compounds contacting with the skin: (1) to assure that no absorption occurs (e.g., certain cosmetics or hazardous substances); (2) to demonstrate that skin absorption takes place (drug delivery); and (3) to test possible toxicity (irritation, corrosion, allergy). Transdermal drug delivery offers an advantageous route of drug administration by eliminating first-pass hepatic metabolism, resulting in fewer systemic side effects [1] and providing sustained drug release for a prolonged period of time. It is a noninvasive route of drug administration, and therefore, offers superior patient compatibility compared to the use of numerous injections. However, skin is the first line of defense of the body and the last barrier separating the organism from the dangerous environment of viruses, pathogens, and toxins. Skin has a very low permeability for foreign molecules across it. A special, hierarchical structure comprising a lipid-rich matrix with embedded developing and dead keratinocytes in the upper layer (15 μm), i.e., the stratum corneum, is mainly responsible for this barrier. In addition to its role as a barrier, both physical and biological, skin is a transport regulator. It regulates the influx and efflux of water molecules into and out of the body. It also makes possible the uptake of a variety of small molecules that are quite lipophilic (partition coefficient, log P > 1.5) and have a low molecular weight, i.e., less than 500 Da [2].

Transporters are transmembrane proteins that are present in a variety of tissues, including the skin. They display broad substrate specificity for endogenous compounds and xenobiotics, and are involved in either the influx or efflux processes [3]. Transporters may therefore strongly impact the distribution of topical drugs or toxic chemicals in the skin, and thus, their pharmacological or toxic effect [4]. A comprehensive set of transporters in various tissues of the human body, including the skin, has been compiled by Bleasby et al. [5]. The ATP binding cassette (ABC), solute carrier (SLC), and organic anion transporting polypeptide (OATP) are the superfamilies of transporters involved in xenobiotic transport in various tissues in the human body. In the ABC families of transporters, multidrug resistance proteins MDR1 and MDR3 and the multidrug resistance-associated proteins MRP1, 3, and 5 through 9 have been identified in the skin [6]. In the SLC family, the following transporter proteins are expressed in human skin: MCT1, OCT 1, PEPT1 and 3, ENT 1, 2, and 4, ATB(0þ), OCTN1 and 2, OAT2, CNT 1–3, PGT, and the OATP-B, -D, -E, -H [6].

A possible role of P-glycoprotein (Pgp) (MDR1) in dermal drug disposition in mouse skin has been demonstrated [3]. The in vitro permeability of rhodamine in the absorptive direction in P-gp-gene knockout mice was 50% to 65% of the permeability, when measured in wild-type mice. In vivo penetration experiments with rhodamine and itraconazole showed that the lack of or inhibition of P-gp in the skin decreased drug distributions therein after topical application. As is the case in the brain, P-gp may be a drug efflux pump from the skin to the blood [3]. Baron et al. used an in vitro model (cell lines) to study how P-gp influences CYP3A4-mediated metabolism. P-gp influences the pharmacokinetics and cytotoxicity of therapeutic drugs. The presence of P-gp was shown to increase the Km values of testosterone 6b- and cortisol 6b-hydroxylase activity [7].

In this study, before the topical administration of P-gp substrates (erythromycin or quinidine), the skin surface was pretreated with a P-gp inhibitor dissolved in dimethylsulfoxide (DMSO), or in parallel, control skins were pretreated with the vehicle (100% DMSO). DMSO is not only a good solvent, but also a penetration enhancer for lipophilic and hydrophilic compounds. DMSO shifts the keratin conformation from an α-helical to a β-sheet structure. It can also interact with intercellular lipids and causes degradation. As DMSO is a perfect solvent, it can alter the drug distribution between the dermal layers and formulation [8].

Although the purposes of skin absorption studies may be different, the models used in such studies are universal. In cases of in vitro/ex vivo skin absorption studies, there are no ethical concerns, while for clinical studies or animal experiments, there are serious restrictions, e.g., testing cosmetics on animals is forbidden [9,10]. The major drawbacks of in vitro skin absorption studies performed using the gold standard, i.e., static Franz diffusion cells, is the absence of capillary blood flow and the large tissue and formulation requirements. In our newly developed skin-on-a chip microfluidic device, microcirculation is mimicked by a dynamic flow-through system [11]. Furthermore, less tissue, drug and additives are needed. Concerning the toxicity testing, the acceptance criteria of in vitro skin absorption data is different by various regulatory organizations. In the European Union, in vitro experiments are accepted for testing skin absorption of pesticides [12,13]. However, NAFTA countries (the USA, Canada, and Mexico (NAFTA 2009)) do not accept in vitro skin absorption data alone without in vivo data for toxicity testing. But due to the ethical restrictions, the majority of the skin penetration data published in the field of cosmetic and pharmaceutical sciences include in vitro/ex vivo dermal absorption studies. Based on the different acceptance requirements of in vitro data, in this study, some in vivo transdermal microdialysis experiments were also conducted to correlate in vitro/ex vivo data of drug penetration with a real, in vivo situation.

There are three different pathways allowing substances to pass through the cutaneous barrier [14]. The first is the intercellular/paracellular penetration pathway, which has been the most relevant one for decades [15]. The second is the follicular/transappendageal penetration pathway [16,17]. The third is the transcellular penetration pathway, i.e., where the substances pass through both the corneocytes, lipid layers and fibroblasts. Intercellular penetration can usually be investigated in vitro or ex vivo, but follicular penetration must be analyzed in vivo because it may evoke the contraction of the pores and ducti during the skin excision process [18].

One of the oldest methods used in penetration studies is the tape stripping method. Tape stripping comprises the successive removal of adhesive films pressed onto the skin. With each adhesive tape, a certain amount of corneocytes are removed from the upper surface of stratum corneum. Usually 10–100 tape strips are used, depending on the species and skin thickness. In this study, excised rat abdominal skins or in vivo tape-stripped rat skin were used. Based on our previous experiments for the optimization of drug concentrations in the collected samples for analytical detection, 10 tape strippings were determined as being optimal for the mechanical sensitization of the penetration surface.

The aims of the current study were: (1) to determine the skin penetration of two P-gp substrate model drugs (quinidine and erythromycin) in the presence or absence of a transporter modulator; (2) to determine whether the freezing of the skin samples had an influence on the drug absorption and P-gp function; (3) to compare the model drug penetration in aged and young rat abdominal skins; (4) to make an ex vivo/in vivo correlation assessment between drug penetration in diffusion cells or in transdermal microdialysis in anesthetized rats; and (5) to test our skin-on-a chip microfluidic system for applicability for transdermal drug delivery and transporter interaction at the dermal barrier.

## 2. Materials and Methods

### 2.1. Model Drugs, Topical Formulations and Other Chemicals

Erythromycin and quinidine were used as P-glycoportein substrate model drugs. Erythromycin was used as a suspension formulation dispersed in a basic cream or as a commercially available cream (Aknemycin^®^), and quinidine was applied as a suspension cream or was fully dissolved in a basic gel.

First, 2 g of erythromycin (Tokyo Chemical Industry Co., Tokyo, Japan) or 2 g of quinidine (Sigma-Hungary Kft., Budapest, Hungary) were dispersed for cream formulation with 4.1 g of liquid paraffin using a mortar and pestle; then, 47 g of white soft paraffin cream (containing polysorbate60 4%, white soft paraffin 26%, liquid paraffin 8 *m*/*m*%, propylene glycol 10%, cetostearyl alcohol 12 *m*/*m*%, and purified water 40%), 10 g propylene glycol and 36.9 g of 0.21% citric acid aqueous solution were added.

The basic gel formulation was kindly donated by Prof. Claudia Mattern (M+P Pharma, Emmetten, Switzerland). In this gel, 2% quinidine (Sigma-Hungary Kft., Budapest, Hungary) was dissolved. The marketed Aknemycin cream (Almirall Hermal GmbH, Reinbek, Germany) was used in a transdermal microdialysis experiment. It contained erythromycin at a concentration of 2%, and the following excipients: liquid paraffin, solid paraffin, cetyl-stearyl-polyglycol-phosphate, cetyl-stearyl-alcohol, 70% sorbit (noncrystal), oleil-oleate, lauryl-polyglycol-phosphate, titan-dioxide, talcum, white vaseline, purified water, and gardenia parfum oil [OGYI-T-2373/01] [19].

For studies executed on the Franz-diffusion cell system and on the skin-on-a-chip microfluidic device, peripheral perfusion fluid (PPF) was used as an extracellular, fluid-like acceptor solution. This was composed of the following components: 147 mM NaCl, 4 mM KCl and 2.3 mM CaCl_2_·2 H_2_O. All substances were acquired from Sigma-Hungary Kft, Budapest, Hungary. Dimethyl-sulfoxide (DMSO) was used as a vehicle for the topically applied PSC-833 (valspodar) due to its good solvent properties and appropriate membrane penetrating ability. Valspodar is a cyclosporin-D analogue, and acts as an inhibitor of P-gp. It was dissolved in DMSO (1 mg PSC-833/1 mL DMSO). Both the DMSO and the PSC-833 were purchased from Sigma-Hungary Kft. (Budapest, Hungary).

### 2.2. Animals

Male Wistar rats (ToxiCoop, Budapest, Hungary) with 250–320 g bodyweight (2.5–3.5 months old) were used for young skin preparation and in vivo transdermal microdialysis experiments, and with 550–700 g bodyweight (17–20 months old) for aged skin preparation. The animals had free access to food and water before the study. The experiments were performed in compliance with the guidelines of the Association for Assessment and Accreditation of Laboratory Animal Care International, and were in accordance with the spirit of the license issued by the Directorate for the Safety of the Food Chain and Animal Health, Budapest and Pest Agricultural Administrative Authority, Hungary (PE/EA/4122-7/2016).

### 2.3. Excision of Skin Samples

Excised skin samples of both young and aged rats were obtained in the same way. Animals were anaesthetized intraperitoneally with chloral hydrate using a dose of 400 mg/kg. Under anesthesia, the hair of the abdominal skin was shaved off with an electric shaver, then epilated by an epilator cream (ISANA^®^ cream from Rossmann, Burgwedel, Germany). Finally, the skin surface was washed and wiped dry. The abdominal skin was then mechanically sensitized 10 times by tape stripping (10 TS) with leucoplast (BSN Medical GmbH, Hamburg, Germany) to remove the upper layers of dead keratinocytes. The removal of the upper skin layer of the stratum corneum by adhesive tape has become a common practice in recent decades [20]. The determination of the kinetics and penetration depth of different drugs by creating concentration–time profiles is facilitated by the use of the noninvasive method of tape stripping [21,22]. Tape stripping makes it easier to evaluate the bioequivalence of topical dermatological dosage forms, and also makes it possible to reach detectable concentrations in the dermis and subcutis in preclinical animal models. Furthermore, the corneocyte layer of the skin does not include active efflux pumps, which would be the targets of our current study. Following these skin preparations, an approximately 1-cm, full-thickness skin incision was made in the caudal part of the abdominal area using scissors. After the first cutting with blunt Metzen scissors, fat, connecting tissue and blood vessels were carefully removed. Then, there were two options for continuing with the preparations, as follows. (1) Fresh skin preparation: Six skin disc-preparations were made from the abdominal, tape-stripped skin (approximately 3 cm in diameter each) for Franz diffusion cell study, or three skin square-preparations (approx. 1.5 cm × 1.5 cm) for skin-on-a-chip experiments. The skin preparations were stored in PPF at room temperature until the initiation of experiments, and were then placed between the donor and acceptor chambers on the cells of the Franz diffusion system or on the chip device. The stratum corneum layer of the skin contacted the test drug formulation, facing the donor chamber, while the dermal/subcutaneous surface contacted the PPF in the acceptor compartments. (2) Frozen skin preparation: Freshly prepared, tape stripped skin preparations were wrapped in an aluminum foil and stored in a deep freezer at −80 °C until the diffusion experiments. On the day of the study, the skin preparations were thawed at room temperature, and then placed on the Franz diffusion cells/chip devices in a similar way as that described for fresh skin preparation.

### 2.4. Franz Diffusion Cell Study

Hanson 6-Cell Manual Diffusion Test System-type Franz diffusion cells were used in the experiments (Hanson, ABL&E-JASCO Hungary Kft, Budapest, Hungary).

**Figure 1:**
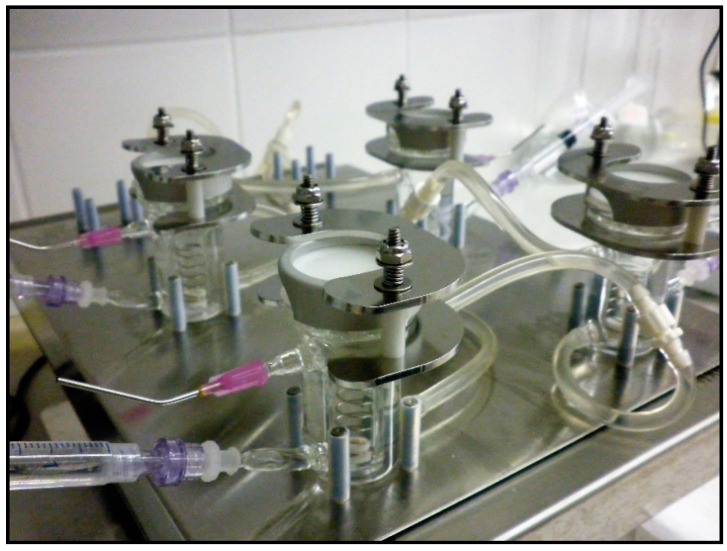
The vertical Franz diffusion cell system with helical magnetic stirrers in the acceptor chamber.

In each unit of the system, there was a heated magnetic stirrer chamber (acceptor compartment), stirred by a helical mixer (Figure 1). A magnet was placed on the bottom of the chamber and the helical mixer was connected to it. The acceptor chambers of the diffusion cells were filled with PPF. On the top of the cells (donor chamber), the skin preparations were placed on a ring (aged or young and frozen or fresh rat abdominal skins) and certain amounts of the tested products (erythromycin or quinidine creams) were applied to measure their penetration. The diffusion surface was 1.767 cm^2^. In the final step, a glass lid was placed onto the donor chambers. In each experiment, six parallel units were used and three cells of them always had the same arrangement with the same conditions. The cells were connected by plastic tubes. In these plastic connectors, distilled water at 32 °C was circulated to mimic the physiological dermal temperature. Ten minutes prior to P-gp substrate treatment, DMSO (control) or PSC-833 dissolved in DMSO was pipetted to the skin surface in a volume of 100 μL. Then, 0.5 g of erythromycin or quinidine cream formulations (2% of each) were placed on the upper surface of the skin and covered with glass lids, and the permeability study was initiated. First, 0.5 mL samples were taken every 30 min from the acceptor compartments. After each sample collection, the removed fluid volume was replaced with PPF. Altogether, 60 samples were taken in each experiment, i.e., 10 from each unit. The collected samples were immediately placed on dry ice and stored at −80 °C until bioanalysis.

### 2.5. Skin-On-A Chip Study

**Figure 2:**
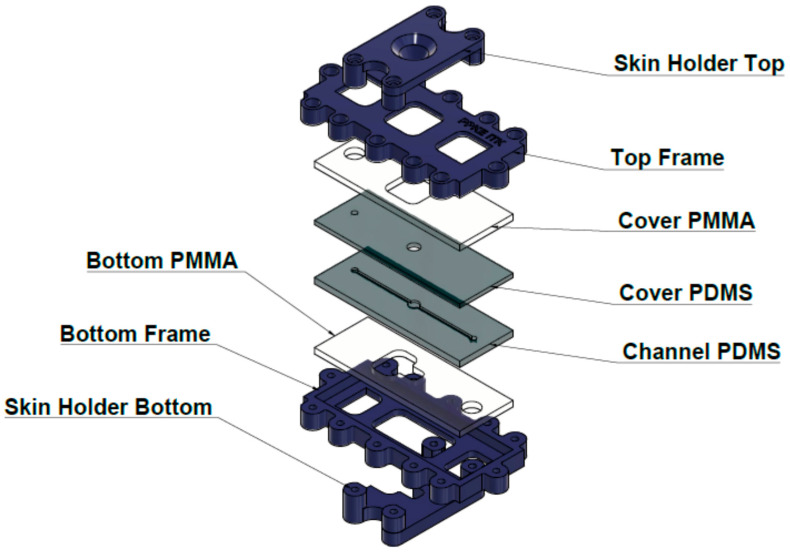
Structure of the “skin-on-a chip” microfuidic diffusion cell. PDMS: polydimethyl-siloxane; PMMA: polymethyl-metacrylate.

Similar to Franz-diffusion cell system, the polydimethylsiloxane-based microfluidic chip is composed of three functional elements: on top there is a donor compartment where the examined formulation was placed, on the bottom there is a receptor compartment, and an integrated skin sample was placed in the middle (Figure 2), as described in detail in our previous paper [11].

The diffusion surface of the skin was 0.50 cm^2^; and it was treated with 1000 µL of the different formulations (gel or cream, 2% of each) using a Microman E piston gel pipette (Gilson, Middleton, WI, USA). The inhibitor solution or DMSO was added to the skin 10 min prior to the application of cream or gel using an automatic pipette at a volume of 100 μL.

**Figure 3:**
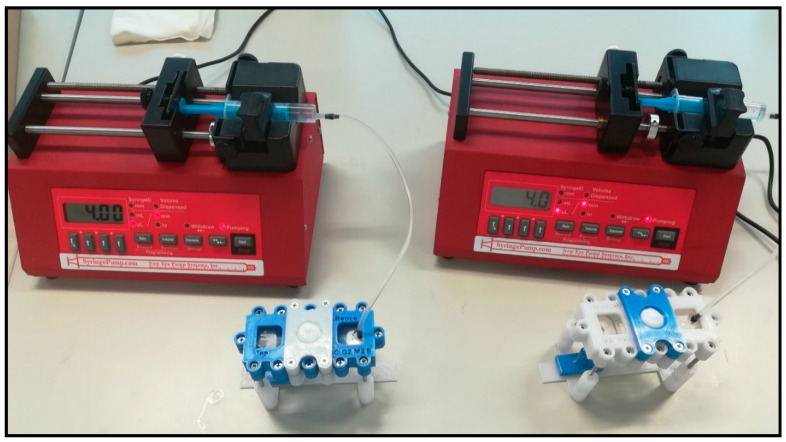
Two simultaneous “skin-on-a chip” experimental setups. The system consists of a programmable syringe pump and a flow-through dynamic microfluidic device. In this photo, the penetration of an active ingredient from a cream formulation across a rat skin preparation was being tested. The collection vials were placed below the devices in the sampling bench.

Contrary to static Franz-diffusion system (and similar to the microdialysis technique), the microfluidic diffusion chamber is a dynamic system, i.e., the flow is continuous below the treated skin surface at the subcutaneous area. The PPF solution was loaded into a 5 mL syringe. Then, the tube was connected to the microfluidic chip, and air bubbles were removed from the syringe, the connected Teflon tubing and from the microchannel of the chip. Flow rate was kept at 4 μL/min during the experiments, controlled by a programmable syringe pump (NE-1000, New Era, Farmingdale, NY, USA). The skin-on-a chip experimental setup is shown on Figure 3. The PPF solution was running through the chip, filling the receptor chamber reservoir and the microfluidic channel and leaving the device at the outlet into collection vials. The samples were collected every 30 min. Collected samples were immediately placed on dry ice and stored at −80 °C until bioanalysis.

### 2.6. Transdermal Microdialysis Study

Microdialysis is a minimally invasive in vivo sampling technique which is used for the continuous monitoring of unbound analyte concentrations in the extracellular milieu in different tissues [23]. In the current study, a transdermal microdialysis technique was applied to investigate the skin penetration of test compounds through the dermal skin layer in anesthetized rats [24].

First, the epilation and shaving of the abdominal skin surface were performed under slight anesthesia, according to the protocol described in the ex vivo studies section.

The experiments were conducted the day after the skin preparation in order to eliminate the possible effect of the epilator cream and the dermal trauma caused by shaving. Animals were reanesthetized intraperitoneally with chloral hydrate with a dose of 400 mg/kg, and fixed in a supine position. In order to keep their body temperature at 37 °C during the experiment, they were placed on a heating pad. The abdominal skin was wiped dry and mechanically sensitized 10 times by tape-stripping with leucoplast (BSN Medical GmbH, Hamburg, Germany).

Two microdialysis probes (MAB11.8.10, Microbiotech, Stockholm, Sweden) were introduced in parallel at a distance of 2 cm through a guide needle (18G) into the dermal tissue. After the withdrawal of the guide needle, the probe membrane stayed in the dermal tissue, the position of which was previously verified by ultrasonic scanning. Probes were then fixed carefully with tape (Omnisilk, Hartmann, Germany). PPF was continuously perfused with a flow rate of 1 μL/min, generated by a programmable syringe pump (CMA 402 Microdialysis Syringe Pump, CMA Microdialysis AB, Kista, Sweden [25]. After a 30-min equilibration period, the first dialysate samples were taken; then, the two transdermal patches (Curatest^®^F, Lohmann Rauscher GmbH, Rengsdorf, Germany) containing 50 mg cream were placed on the skin surface, exactly above the microdialysis probe membranes. Thereafter, samples were collected into 300 µL collecting vials every 30 min for 5 h. The collected dialysate samples were placed on dry ice and stored at −80 °C until bioanalysis.

### 2.7. Erythromycin and Quinidine Sample Analysis by LC-MS/MS

The identification of the quinidine and erythromycin concentration in the samples was performed on a Sciex 3200QTrap hybrid tandem mass spectrometer coupled to Perkin Elmer Series 200 HPLC system. Electrospray ionization was used in positive ion detection mode with MRM transitions of quinidine, as follows: 325.2/307.2 (quantifier) and 325.2/172 (qualifier), and for erythromycin, 734.2/158.1 (quantifier) and 734.2/576.4 (qualifier), with collision energies of 31 V, 45 V, 37 V and 29 V respectively. The dwell time of the transitions was 300 msec. Source conditions were as follows. Curtain gas: 35 arbitrary unit (au); spray voltage: 5000 V; source temperature: 450 °C; nebulizer gas: 40 au; drying gas: 40 au; and declustering potential: 20 V. The samples were introduced to the system via an HPLC system consisting of a binary pump, an autosampler and a column compartment unit. A Phenomenex Synergi Fusion RP column (50 v 2 mm, 4 µm, 80 Å) column was applied for the separation using 0.1% formic acid in water as eluent A and 0.1% formic acid containing acetonitrile as eluent B in gradient elution mode. The gradient started at 85% of eluent A, and eluent B was increased to 90% by 3 min and kept at that concentration for 0.5 min, before being decreased to the initial composition by 0.3 min and kept there for 2.2 min. The overall run time was 6 min. Next, 5 µL of samples were injected. The column was kept at ambient temperature. Two separate calibration regions were used to achieve precise identification. The lower concentrations were quantitated in the range of 1–125 ng/mL, and the higher concentrations in the range of 125–20,000 ng/mL.

### 2.8. Statistical Analysis

The data were given as means +/− standard error (SE). The different treatment groups were compared by Student *t*-test (2,2) in the cases of AUC and Cmax values, and with ANOVA followed by Tukey multiple comparison test in the cases of concentration-time profiles for analyzing the effect of more factors (aging, PSC-833 treatment). A statistical analysis was generated using Microsoft Excel 2016 or OriginLab 8.0, (Macasoft Bt, Győr, Hungary).

## 3. Results

### 3.1. Skin Penetration in Static Franz Diffusion Cells

**Figure 4:**
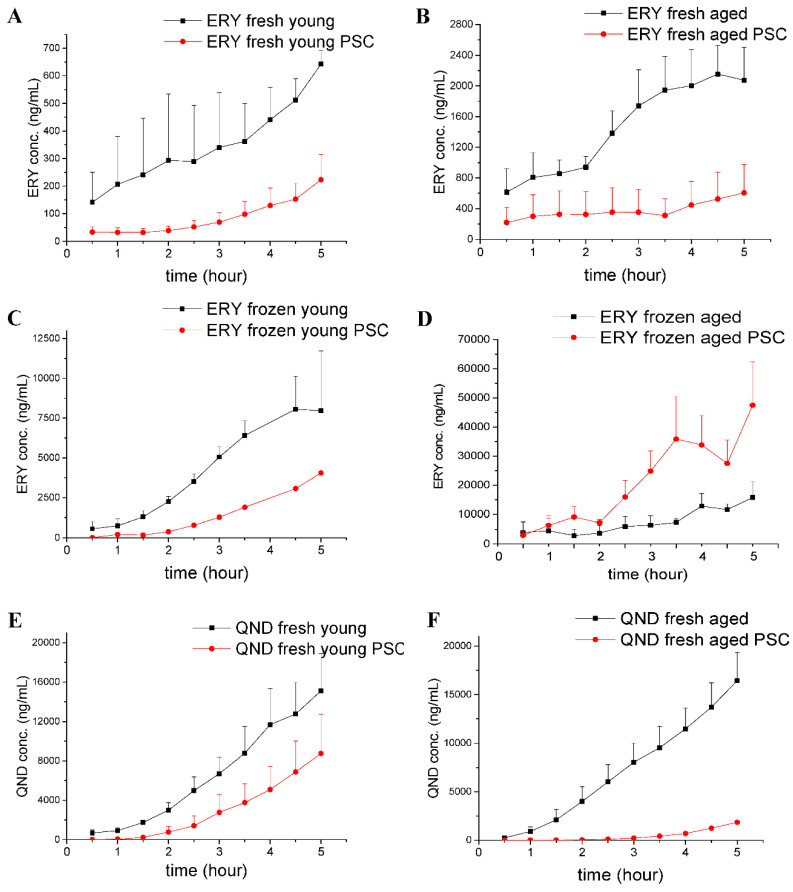
Concentration-time profiles determined in Franz diffusion cells in freshly prepared (**A**,**B**,**E**,**F**) and frozen (**C**,**D**) rat skin samples. Erythromycin (ERY) absorption was measured (**A**–**D**) in young (**A**,**C**) and aged (**B**,**D**) skin preparations. Quinidine (QND) absorption was measured (**E**,**F**) in young (**E**) and aged (**F**) skins. Both ERY and QND were applied as a 2% cream. The black lines show the groups where the skins were pretreated with DMSO followed by ERY or QND treatment. The red lines show the groups where the skins were pretreated with PSC-833 containing DMSO followed by ERY or QND treatment. *n* = 3. The statistical analysis of generated AUC and Cmax data is presented in Figure 5 and Figure 6.

**Figure 5:**
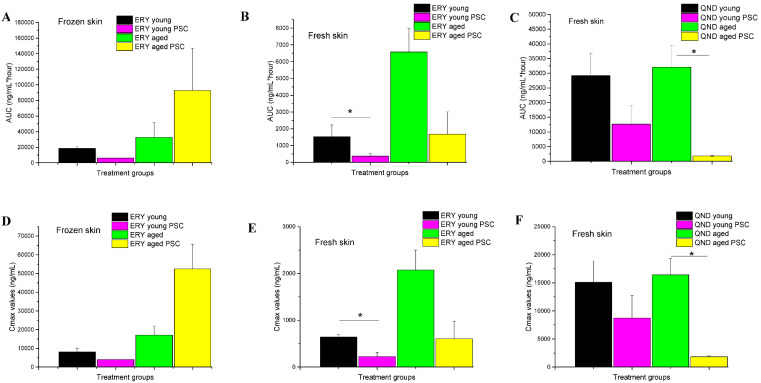
Ex vivo skin penetration of erythromycin (ERY) and quinidine (QND) measured in frozen (**A**,**D**) and fresh (**B**,**C**,**E**,**F**) rat abdominal skin preparations in the presence or absence of topical PSC-833 (valspodar) pretreatment in Franz diffusion cells. Data are presented as means +/− SE. *n* = 3. Y: young rat skin, A: aged rat skin. * *p* < 0.05 by Student *t*-test (2,2).

**Figure 6:**
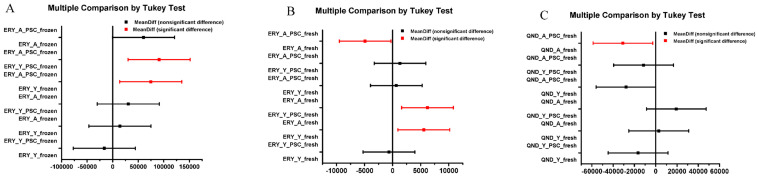
Multiple comparison of means of area under the concentration-time curves (AUC-s) of P-gp substrate penetration across the dermal barrier by Tukey test. (Panel **A**) Aged and young, frozen rat skin samples were compared for erythromycin (ERY) penetration in the presence or absence of PSC-833 pretreatment. (Panel **B**) Aged and young, freshly prepared rat skin samples were compared for erythromycin (ERY) penetration in the presence or absence of PSC-833 pretreatment. (Panel **C**) Aged and young, freshly prepared rat skin samples were compared for quinidine (QND) penetration in the presence or absence of PSC-833 pretreatment.

Using Franz diffusion cell experiments, we attempted to answer the following questions. First, we wanted to see whether the frozen skins and the freshly prepared skins could be used to study efflux transporter functionality in the dermal barrier. The results of these comparative experiments are shown in Figure 4A–D. Secondly, we wanted to test whether the effect of skin aging, with its anatomical, physiological and physical consequences, has an influence on P-gp function (Figure 4A vs. Figure 4B; Figure 4C vs. Figure 4D; Figure 4E vs. Figure 4F). Finally, we tested the effect of P-gp modulation by PSC-833 on the absorptive transport of both ERY (Figure 4A–D) and QND (Figure 4E,F) through the dermal barrier. The AUC and C_max_ values calculated from concentration–time curves are shown in Figure 5A–F. The data were statistically analyzed by the comparison of AUC and Cmax values using a Student *t*-test (Figure 5) and ANOVA, followed by a Tukey test, as shown in Figure 6A–C.

In all experiments using the Franz diffusion cells, cumulative time–concentration profiles were seen (Figure 4). The drug penetrations were higher in the controls (treated with DMSO+P-gp substrate) than in the PSC-833 pretreated skins, proving an absorptive direction of P-gp-mediated transport in the dermal barrier. These findings were consistent in all freshly prepared skins (Figure 4A,B,E,F and Figure 5B,C,E,F). However, in the frozen tissues, a difference was detected between the young and aged preparations. In the young skin, the P-gp inhibitor caused a similar reduction in the absorption to the fresh preparations, while in the aged skins, the opposite effect was detected (Figure 4C,D and Figure 5A,D). Furthermore, in both fresh and in frozen samples, the permeability of aged skins was much higher than that of the young skins (Figure 4A vs. Figure 4B and Figure 4C vs. Figure 4D). This can be explained by the reduced thickness and lower collagen, water and extracellular matrix contents of aging skin. Additionally, it was observed that the diffusion of erythromycin through the frozen/thawed tissues was enhanced compared to fresh skins. This can be the consequence of structural changes, i.e., extension of the pore size, in the epidermis/dermis due to the freezing process. Concerning the pumping function of P-gp, it seemed to be broken in the aged frozen skins and reduced in the young frozen skins due to the complete or partial functional and/or structural degradation of this transporter protein.

In the quinidine penetration experiments (Figure 4E,F and Figure 5C,F), P-gp inhibition resulted in a higher reduction in absorption in aged than in young skins; however, the permeability of the controls was found to be independent of age. These data suggest that in the transdermal transport of quinidine, P-gp plays a specific role, and probably other mediating factors became less important or were downregulated with advanced age. In contrast, erythromycin permeability was increased in the aged skin samples, which be evidence of a role of factors other then P-gp in the transdermal penetration of this molecule (e.g., increased porosity, more expressed role of transappendageal, follicular pathway).

The effect of topical inhibitor pretreatment and age on the drug permeability of the skin tissues was statistically compared for three separate groups (Figure 6): (1) Frozen skins, ERY cream; (2) fresh skins, ERY cream; (3) fresh skins, QND cream. The AUC values were analyzed by ANOVA followed by Tukey test. In frozen, ERY treated skins, only the permeability of PSC-833 pretreated aged and young skins, and the PSC-833 pretreated aged and control young skins, differed in a statistically significant manner (Figure 6A). This results indicate that the freezing process caused changes in P-gp functionality, both in aged and in young skins. In fresh, ERY treated skins, PSC-833 had a significant effect on permeability, both in young and aged skins, and the young and old control skins also differed significantly (Figure 6B). In fresh QND-treated skins, PSC-833 had a statistically relevant effect only on aged subjects (Figure 6C).

### 3.2. Skin Penetration in Dynamic “Skin-On-A-Chip” Device

A 2% suspension cream of quinidine was used for Franz diffusion cell study, while for the microfluidic chip, to reach proper confluency and to avoid inhomogeneity and air bubbles on the skin surface, quinidine was applied via a 2% gel formulation.

**Figure 7:**
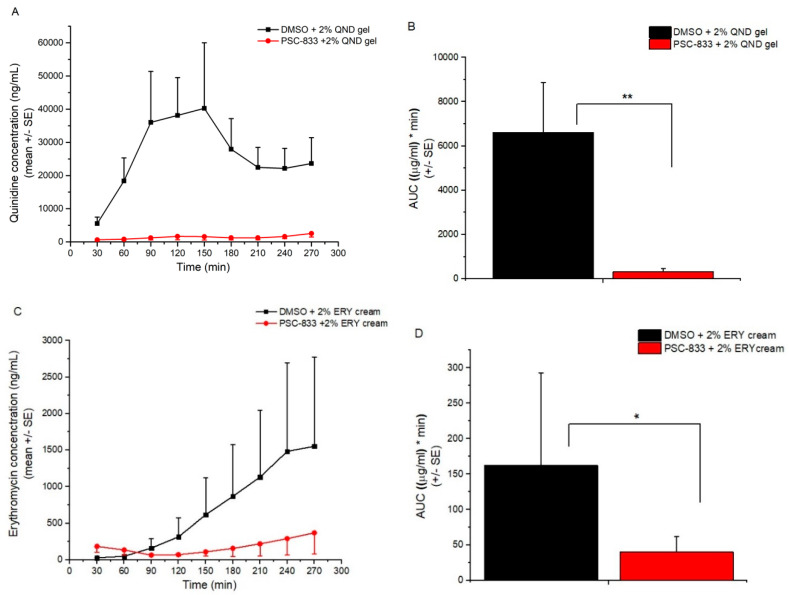
Skin penetration of quinidine gel (QND) (Panel **A**,**B**) and erythromycin cream (ERY) (Panel **C**,**D**) across the freshly prepared, excised, young rat abdominal skin on the microfluidic chip. Concentration-time profiles (Panel **A**,**C**) and AUC values (Panel **B**,**D**) are shown as means +/− SE, *n* = 3–5. Statistical analysis was performed by Student *t*-test (2,2) * *p* < 0.05, ** *p* < 0.01.

In the microfluidic device, the skin penetration of quinidine from gel followed a three-phase profile: first, an absorption period (0–90 min), then a plateau phase (90–180 min), and finally, an elimination phase (Figure 7A). The effect of P-gp inhibition is highly significant, and results in a dramatic reduction in transdermal delivery. The dynamics of erythromycin absorption from cream is different (Figure 7C). In the 270-min observation period, only the absorption phase could be reached, or the plateau was initiated. One possible explanation for this, beside the excursive molecular weights (Mw_QND = 324.4, Mw_ERY = 733.9) and lipophilicity (logP_QND = 3.44, logP_ERY = 3.06), may be the difference in the formulation of the two drugs (gel and suspension cream). However, P-gp-mediated transport was clearly demonstrated for both drugs.

### 3.3. Skin Penetration In Vivo

**Figure 8:**
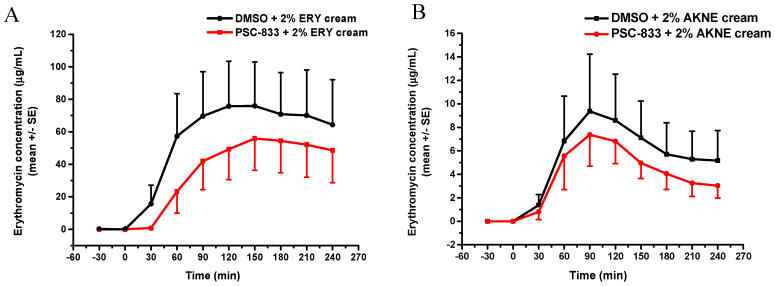
Concentration-time profiles of the absorption of topical erythromycin cream (ERY) or Aknemycin^®^ cream measured by transdermal microdialysis in vivo in anesthetized rats. (Panel **A**) 2% ERY paraffin-based cream, (Panel **B**) AKNE: 2% erythromycin containing Aknemycin^®^ cream. *n* = 5/group.

To verify the influence of topical P-gp inhibition on the penetration of P-gp substrate into the deeper layers of the skin, in vivo transdermal microdialysis was conducted. The model drug was erythromycin, and two cream formulations were compared (paraffin-based and Aknemycin). Based on the results (Figure 8A,B), the drug release and penetration was higher and longer lasting from the paraffin-based cream than from the Aknemycin cream. In case of Aknemycin, the peak absorption was detected at 90 min after drug administration, and then it started to be slowly eliminated from the subcutaneous tissue. The P-gp modulation caused a moderate but reproducible reduction in drug absorption.

## 4. Discussion and Conclusions

In the current study, the delivery of efflux transporter substrates through the dermal barrier was tested by different ex vivo and in vivo techniques, and some methodological conditions were optimized. In each methodological situation, a mechanical penetration enhancer technique, i.e., adhesive tape stripping, was applied. This method and also the cyanoacrylate stripping are traditional techniques, but they were recently described and analyzed by Dong and coworkers [26]. These ways of mechanical sensitization are widely used in the literature to disrupt the barrier function of the stratum corneum by making the dead keratinocyte layer thinner, and thereby, improving drug absorption [27,28,29]. This method is also used for sampling the stratum corneum and testing the depth of the penetration of the molecule into the skin (both in human and animal skins). In such cases, the tapes are subjected to analysis for quantitation of the absorbed molecules at each removed layer of the epidermis. The process is repeated, and in this way, it becomes possible to track drug penetration (in terms of both degree and depth). In our case, the aim of the mechanical sensitization was to increase the drug penetration; as P-gp is not functional in the outermost layer of the stratum corneum, the removal of these cells did not influence drug-drug interactions in our experiments.

Unwanted side effects are a frequent risk associated with the systemic administration of multidrug medication. The modulation of a target protein (e.g., P-gp or a CYP enzyme) may lead to multiplication of drug exposure at different organs [30,31]. Erythromycin is metabolized by enzymes of the cytochrome P450 family, in particular, by isozymes of the CYP3A superfamily [32]. If other CYP3A substrates are taken together with erythromycin, the substrate levels increase, causing adverse effects. Several drug interactions involve erythromycin, resulting in increased drug exposure and potential unexpected side effects. Some drugs used to treat migraines are also CYP3A4 substrates, and their adverse effects may be more pronounced if erythromycin is coadministered [33]. A case report described a correlation between erythromycin and ventricular tachycardia due to coadministration with drugs that prolong its metabolism [34].

Quinidine is an inhibitor of the cytochrome P450 enzyme 2D6, and a substrate, but also an inhibitor of the transport protein P-glycoprotein. Quinidine can cause some peripherally acting drugs, such as loperamide, to exert central nervous system side effects, like respiratory depression in cases of cotreatment [35]. Many preclinical studies have provided also evidence on the increased brain exposure to transporter substrates due to drug–drug interactions at the efflux pumps, like P-gp, located at the blood-brain barrier [30,36,37,38]. However, to date, no study has reported on drug–drug interactions between topically applied drugs. The current experiments provide evidence of changed absorption and the risk of interactions or adverse events in cases when both therapeutic agents are coadministered topically and act on the same transport protein, even though the topical penetration to the systemic circulation is usually not very high. The next question to be answered is whether a systemic and topical P-gp substrate may have a distinct interaction or change pharmacokinetic profile in cases of coadministration.

In conclusion, in this study, the authors successfully demonstrated that (1) P-gp is functional in the skin, both in vivo and ex vivo, and that the direction of its transport is absorptive, in accordance with the findings of Hashimoto et al., 2017 [39]; (2) the freezing of the excised skin samples, however, impairs the P-gp function and increases tissue permeability; therefore, frozen tissues are not recommended for use in transporter studies; (3) it was earlier reported that the thickness and physicochemical properties of skin change with age; this study contributed to these data by providing results on the maintained P-gp function also in aged skins; (4) the inhibitory effect of PSC-833 on the absorption of both ERY and QND in the dermal barrier was shown ex vivo in diffusion cells, and also in a dynamic skin-on-a-chip device. The same tendency can be seen in vivo, but in this multifactorial environment, the effect is not as significant as it is ex vivo; (5) the utilization is described of a new skin-on-a-chip microfluidic device for the investigation of skin penetration and transporter interactions at the dermal barrier.

The water content and physicochemical properties of the excised skins may have an influence on drug permeability. In this study, this question was not the focus. However, our future plan is to perform a physicochemical characterization and comparison of different skin samples by measurement of transepithelial water loss (TEWL), pH and sebum at the skin surface. Furthermore, the interaction between systemic and topical drugs should be tested in vivo, and human reconstructed, full-thickness skins should also be investigated and integrated into microfluidic devices to assess their utility in in vitro dermal barrier experiments.
